# Pathologie cutanée du sujet âgé en dermatologie à Lomé, Togo: étude de 325 cas

**DOI:** 10.11604/pamj.2014.18.151.3066

**Published:** 2014-06-17

**Authors:** Koussake Kombaté, Bayaki Saka, Abas Mouhari-Toure, Raymond Kossi Barruet, Waguena Gnassingbé, Séfako Akakpo, Akouavi Maboudou, Dadja Essoya Landoh, Kissem Tchangaï-Walla, Palokinam Pitché

**Affiliations:** 1 Service de Dermatologie, CHU Sylvanus Olympio, Université de Lomé, Lomé, Togo; 2 Consultation de Dermatologie, Clinique Barruet, Lomé, Togo; 3 Cabinet Médical Saint Michel, Lomé, Togo; 4 Division de l'Epidémiologie, Ministère de la santé, Togo

**Keywords:** Pathologie cutanée, sujet âgé, Lomé (Togo), skin disease, elderly, Lomé

## Abstract

**Introduction:**

Les pathologies cutanées du sujet âgé ont des manifestations cliniques qui diffèrent de celles du sujet jeune. Le but de cette étude était de déterminer les pathologies cutanées motivant une consultation du sujet âgé en dermatologie à Lomé (Togo).

**Méthodes:**

Il s'agissait d'une étude transversale descriptive portant sur les nouveaux diagnostics d'affections cutanées colligés entre le 1er janvier 2010 et le 31 décembre 2011 chez les sujets âgés de 65 ans et plus en dermatologie à Lomé.

**Résultats:**

Durant la période d’étude, 325 patients âgés de 65 ans et plus (2,4%) ont été examinés sur l'ensemble des 13440 patients ayant consulté en dermatologie. Ces 325 patients ont consulté pour 375 nouveaux diagnostics. L’âge moyen des patients était de 72,8 et le sex-ratio (H/F) de 0,8. Les mycoses représentaient le principal motif de consultation (18,7%), suivies par l'eczéma (17,9%), le prurit (13,1%), les tumeurs (9,0%) dont 76,5% de tumeurs bénignes et les troubles de la kératinisation (5,1%). Les ulcères de jambe étaient observés chez 2,1% des patients. Les mycoses étaient essentiellement représentées par les intertrigos (52,9%) et les dermatophyties de la peau glabre (25,7%). La maladie de Kaposi et les chéloïdes représentaient respectivement les tumeurs malignes et bénignes les plus fréquentes.

**Conclusion:**

Cette étude montre une fréquence élevée des mycoses, de l'eczéma et du prurit chez les sujets âgés à Lomé. La fréquence élevée des mycoses pourrait s'expliquer par les conditions climatiques (chaleur, humidité) et celle du prurit par le vieillissement de la peau.

## Introduction

Les pathologies cutanées du sujet âgé sont variées et constituent une véritable préoccupation pour les médecins en termes de diagnostic, de prise en charge et de suivi [1]. Leur manifestation clinique n'est pas classique et diffère de celle du sujet jeune [2]. De nombreuses études ont été réalisées dans de nombreux pays [3–6], pour déterminer le profil de ces affections chez le sujet âgé, mais aucune de ce genre n'a encore été menée au Togo. Le but de cette étude était de documenter le profil de la pathologie cutanée chez le sujet âgé en dermatologie à Lomé (Togo).

## Méthodes

Une étude transversale et multicentrique a été réalisée entre janvier 2010 et décembre 2011 dans 3 structures publiques (services de dermatologie des centres hospitaliers universitaires Sylvanius Olympio et Campus, centre de dermatologie de Gbossimé) et dans 3 cabinets privés de dermatologie de la ville de Lomé. Seuls les patients âgés de 65 ans et plus qui étaient reçus en dermatologie ont été inclus dans la présente étude. Pour chaque patient, les dermatologues ont recueilli sur une fiche d'enquête validée, l’âge, le sexe, la durée d’évolution de la pathologie cutanée avant la consultation, le ou les nouveaux diagnostics.

### Analyse statistique

La description simple de l′échantillon a été possible grâce au calcul des proportions ou des moyennes selon que la variable soit catégorielle ou numérique. Toutes les analyses ont été faites en utilisant le logiciel Epi Info version 3.5.1.

### Considération éthique

Nous avons obtenu le consentement éclairé des patients avant d’être inclus dans cette étude. Les objectifs et les résultats attendus de l’étude ont été expliqués aux patients

## Résultats

Au cours de la période d’étude, 325 (2,4%) des 13440 patients reçus en consultation étaient âgés 65 ans et plus. Ces 325 patients ont consulté pour 375 nouveaux diagnostics (42 patients ont consulté pour 2 nouveaux diagnostics et 4 patients pour 3 nouveaux diagnostics). L’âge de nos patients variait entre 65 et 95 ans (moyenne: 72,8 ans et écart-type: 6,9 ans). La majorité d'entre eux (64,3%) avaient un âge compris entre 65 et 74 ans. Ils se répartissaient en 127 hommes et 198 femmes, et cette prédominance féminine était notée dans toutes les tranches d’âge. Les pathologies cutanées diagnostiquées sont présentées dans le Tableau 1. Les mycoses, au 1er rang des dermatoses d'origine infectieuse et de l'ensemble des dermatoses, touchaient le sexe féminin de manière prépondérante (sex-ratio H/F=0,3) et étaient essentiellement représentées par les intertrigos (52,9%), les dermatophyties de la peau glabre (25,7%) et les onychomycoses (11,4%). Les dermatoses d'origine bactérienne touchaient également le sexe féminin de manière prépondérante (sex-ratio H/F=1,7) et étaient représentées par les pyodermites (44,5%), l’érysipèle (33,3%) et la lèpre (22,2%). Le zona était la dermatose d'origine virale la plus fréquente (68,8%) et correspondait à 2,9% de toutes les dermatoses. Les deux cas de dermatoses d'origine parasitaire étaient la gale. Un eczéma était diagnostiqué dans 67 cas avec une prédominance féminine (sex-ratio H/F=0,8). Le prurit était sénile dans 41 cas et associé dans 8 cas à une xérose cutanée. Le xérose cutanée isolée, sans prurit était notée dans 6 cas. Notre série comportait 34 cas de tumeurs dont les plus fréquentes étaient les tumeurs bénignes représentées principalement par les chéloïdes (42,3%), les kystes (26,9%) et les molluscums pendulum (11,5%). Les tumeurs malignes étaient représentées par 6 cas de maladie de Kaposi (1,9% de toutes les dermatoses) dont 4 formes épidémiques et 2 formes africaines, un cas de carcinome épidermoïde et un cas de mélanome malin de type acral chez une femme de 70 ans. Les troubles de la kératinisation étaient représentées par les kératodermies palmo-plantaires (42,1%), le psoriasis (31,6%) et les durillons (26,3%). Les troubles pigmentaires étaient essentiellement représentés par le vitiligo (75%) et l'urticaire aigue était le type d'urticaire le plus fréquent (66,7%). Six cas de toxidermies ont été notés dont 5 cas d’érythème pigmenté fixe et un cas de nécrolyse épidermique toxique. Les ulcères de jambe touchaient de manière prépondérante les hommes (sex-ratio H/F=1,7). Ces derniers évoluaient depuis en moyenne 3 ans. Les connectivites étaient représentées par la sclérodermie (2 cas) et le lupus érythémateux chronique (2 cas). Enfin, trois cas de dermatoses bulleuses auto-immunes dont 2 cas de pemphigoïde bulleuse et un cas de pemphigus vulgaire ont été notés.

**Tableau 1:** Dermatoses chez le sujet âgé de 65 ans et plus

	Nombre de cas	Fréquence (%)
**Dermatoses d'origine infectieuse**	115	30,7
Dermatoses d'origine mycosique	70	18,7
Dermatoses d'origine bactérienne	27	7,2
Dermatoses d'origine virale	16	4,3
Dermatoses d'origine parasitaire	2	0,5
Eczéma	67	17,9
Prurit	49	13,1
Tumeurs	34	9,0
bénignes	26	6,9
malignes	8	2,1
Troubles de la kératinisation	19	5,1
Xérose cutanée	14	3,7
Prurigo	10	2,7
Ulcère de jambe	8	2,1
Troubles pigmentaires	8	2,1
Urticaire	6	1,6
Toxidermies	6	1,6
Erythème pellagroïde	5	1,3
Connectivites	4	1,1
Dermatoses bulleuses auto-immunes	3	0,8
Autres	27	7,2
**Total**	**375**	**100**

## Discussion

Dans cette étude, les motifs de consultation du sujet âgé étaient dominés par des affections chroniques ou réellement pathologiques, et très peu de problèmes esthétiques. Le nombre de consultations rapporté dans notre étude est faible, ce qui ne permet pas d'extrapoler nos résultats à l'ensemble des personnes âgées à Lomé et encore moins dans le pays. Par ailleurs, les services de dermatologie n'ont pas l'exclusivité de la prise en charge des maladies de la peau, ce fait couplé aux problèmes d'accessibilité financière des populations aux soins expliquent en grande partie le faible nombre de consultations observées au cours de la période d’étude. En effet, compte tenu du contexte socio-économique, culturel et sanitaire, couplé à la bénignité de certaines affections cutanées, il est très probable que de nombreux patients aient recours à des traitements traditionnels ou à l'automédication. Malgré ces limites, cette étude nous a permis de documenter les principaux motifs de consultations du sujet âgé à Lomé. La proportion des sujets âgés dans notre étude représente moins de la moitié de celles rapportées en Tunisie [3, 4], et moins du quart de celle rapportée en Taïwan [5]. Ces résultats peuvent être expliqués par la faible espérance de vie en Afrique subsaharienne liée à la pauvreté et à la sous-médicalisation. Au Togo par exemple, la proportion des sujets âgés représentait 3,9% à la suite du dernier recensement de la population en 2010.

Notre série montre une petite différence entre les dermatoses observées chez le sujet âgé et celles de la population générale vue en dermatologie. Les mycoses, l'eczéma et le prurit étaient au premier rang des dermatoses chez le sujet âgé alors que dans la population générale, il s'agissait des eczémas, des mycoses et des acnés [7]. La forte proportion du prurit chez le sujet âgé est liée à l’âge des patients étudiés (forte proportion du prurit sénile qui était un diagnostic d’élimination) au vieillissement de la peau et à la xérose cutanée. En revanche, l'acné est une dermatose de l'adolescence, ce qui explique sa troisième place dans les motifs de consultation de la population générale togolaise qui est à prédominance de sujets jeunes. Comme dans notre étude, les mycoses constituaient le premier motif de consultation des sujets âgés en Tunisie [3, 4]. Contrairement à notre étude et aux études Tunisiennes où les mycoses cutanées étaient au premier plan suivies de l'eczéma, les séries occidentales montraient une prédominance de l'eczéma suivi des tumeurs (Tableau 2). La fréquence élevée des mycoses dans notre série et dans les séries tunisiennes pourrait être expliquée par les facteurs climatiques en Afrique qui associent chaleur et humidité. Mais, la faible proportion de la gale dans notre étude comparativement aux séries tunisiennes peut être expliquée par l'absence au Togo de maisons de retraite où la gale évolue par épidémie. Les tumeurs malignes sont diagnostiquées à une fréquence très faible dans notre étude contrairement aux autres séries [3–5, 8–10]. Cette différence est liée au fait que notre étude a porté sur des sujets à peau génétiquement pigmentée qui ont un phototype foncé dont la mélanine épidermique plus dense agit comme un écran face aux rayons ultraviolets [11]. Aussi, la première tumeur maligne observée dans notre était la maladie de Kaposi alors que les carcinomes basocellulaires étaient les premiers cancers cutanées dans les autres séries [3–5]. La maladie de Kaposi est devenue le cancer cutané le plus fréquent actuellement en Afrique subsaharienne devant les carcinomes cutanés et le mélanome [12, 13]. Cette fréquence inhabituelle de ce cancer cutané est liée à la prévalence élevée de l'infection à VIH dans cette partie du monde [14]. Parmi les tumeurs bénignes, les chéloïdes occupaient la première place contrairement aux autres séries où la première place était occupée par les kystes [3], ou par des kératoses séborrhéiques [4].

**Tableau 2:** Principales dermatoses du sujet âgé selon les séries

	Notre étude Lomé	Tunisie Tunis [3]	Tunisie Sfax [4]	Croatie Karlovac [8]	Canada Ottawa [9]	USA New York [10]
Mycoses (%)	18,7	16,9	16	6,81	3,4	4,5
Eczéma (%)	17,9	11,9	12	7,30	16,3	37,9
Prurit (%)	13,1	6,4	4,2	6,20	1,2	1,8
TB* (%)	6,9	8,0	14,7	18,98	13,8	11,5
TM** (%)	2,1	3,0	12,3	9,37	12,6	9,0

* TB: Tumeurs bénignes

** TM: tumeurs malignes

## Conclusion

Cette étude montre une faible proportion des sujets âgés de 65 ans et plus en dermatologie à Lomé. Par ailleurs, elle confirme que les mycoses cutanées sont les affections cutanées les plus fréquentes chez le sujet âgé en Afrique. Cette fréquence élevée des mycoses pourrait s'expliquer par les conditions climatiques qui associent chaleur et humidité.
